# Multimodal apparent diffusion MRI model in noninvasive evaluation of breast cancer and Ki-67 expression

**DOI:** 10.1186/s40644-024-00780-x

**Published:** 2024-10-11

**Authors:** Huan Chang, Jinming Chen, Dawei Wang, Hongxia Li, Lei Ming, Yuting Li, Dan Yu, Yu Xin Yang, Peng Kong, Wenjing Jia, Qingqing Yan, Xinhui Liu, Qingshi Zeng

**Affiliations:** 1 Department of Radiology, Shandong Provincial Qianfoshan Hospital, Shandong University, No.16766 Jingshi Road, Jinan, Shandong China; 2 Department of Radiology, The First Affiliated Hospital of Shandong First Medical University & Shandong Provincial Qianfoshan Hospital, Jinan, Shandong China; 3 Department of Radiology, The Second Hospital of Shandong University, Jinan, Shandong China; 4 Department of Radiology, The First College of Clinical Medicine, Shandong University of Traditional Chinese Medicine, Jinan, Shandong China; 5 United Imaging Research Institute of Intelligent Imaging, Beijing, People’s Republic of China; 6 Department of Breast Surgery, The First Affiliated Hospital of Shandong First Medical University & Shandong Provincial Qianfoshan Hospital, Jinan, Shandong China; 7 Department of Radiology, The First Affiliated Hospital of Shandong First Medical University & Shandong Provincial Qianfoshan Hospital, Shandong First Medical Universityand, Shandong Academy of Medical Sciences , Jinan, Shandong China

**Keywords:** Diffusion magnetic resonance imaging, Breast cancer, Multimodal apparent diffusion analysis

## Abstract

**Background:**

To assess the capability of multimodal apparent diffusion (MAD) weighted magnetic resonance imaging (MRI) to distinguish between malignant and benign breast lesions, and to predict Ki-67 expression level in breast cancer.

**Methods:**

This retrospective study was conducted with 93 patients who had postoperative pathology-confirmed breast cancer or benign breast lesions. MAD images were acquired using a 3.0 T MRI scanner with 16 b values. The MAD parameters, as flow (f_F_, D_F_), unimpeded (fluid) (f_UI_), hindered (f_H_, D_H_, and α_H_), and restricted (f_R_, D_R_), were calculated. The differences of the parameters were compared by Mann–Whitney U test between the benign/malignant lesions and high/low Ki-67 expression level. The diagnostic performance was assessed by the area under the receiver operating characteristic curve (AUC).

**Results:**

The f_R_ in the malignant lesions was significantly higher than in the benign lesions (*P* = 0.001), whereas the f_UI_ and D_H_ were found to be significantly lower (*P* = 0.007 and *P* < 0.001, respectively). Compared with individual parameter in differentiating malignant from benign breast lesions, the combination parameters of MAD (f_R_, D_H_, and f_UI_) provided the highest AUC (0.851). Of the 73 malignant lesions, 42 (57.5%) were assessed as Ki-67 low expression and 31 (42.5%) were Ki-67 high expression. The Ki-67 high status showed lower D_H_, higher D_F_ and higher α_H_ (*P* < 0.05). The combination parameters of D_H_, D_F_, and α_H_ provided the highest AUC (0.691) for evaluating Ki-67 expression level.

**Conclusions:**

MAD weighted MRI is a useful method for the breast lesions diagnostics and the preoperative prediction of Ki-67 expression level.

**Supplementary Information:**

The online version contains supplementary material available at 10.1186/s40644-024-00780-x.

## Introduction

Among females, breast cancer is the most common cancer and the leading cause of cancer death, with the incidence rate rising globally [1]. The expression of Ki-67, a key marker of cellular proliferation, plays an important role in the molecular subtype classification and subsequent therapy selection in breast cancer [2–4]. Breast cancer with a high Ki-67 status is more likely to be heterogeneous and aggressive, and have a higher risk of recurrence [5]. Therefore, it is crucial to measure the Ki-67 index before selecting a specific therapy for patients with breast cancer. Biopsy has certain limitations, and therefore devising diagnostic biomarkers for breast lesion characterization can offer additional information to clinical biopsy. The ability to non-invasively track microscopic tissue modifications over time offers significant potential in assessing surrogate indicators of disease response or progression.

Through its use of endogenous water, diffusion weighted imaging (DWI) can be used to probe local tissue structure to infer the whole tumor information [6]. DWI exhibits a remarkable sensitivity to the displacement of water particles at length scales that are substantially smaller than the achievable image resolution. This sensitivity is particularly significant as water mobility is inherently influenced by its intricate interactions with cellular structures. Most studies have studied the monoexponential diffusion model, which is based on the monoexponential Gaussian linear model [7–9]. The lack of oxygen and nutrient supply, coupled with the occurrence of inflammation and angiogenesis, can lead to necrosis, cyst formation, and hemorrhage in breast lesions, which resulting the increased heterogeneity of breast lesions [10–12]. Voxels may contain various combinations of milieus, each with varied tissue properties, especially in heterogeneous pathology, such as cellularity, vascularity, cytotoxic and vasogenic edema, fluidity, perfusion status. Due to the complex microstructure, each voxel may contain a wide and non-uniform distribution of diffusion process, undermining the use of apparent diffusion coefficient (ADC) to specifically describe tissue properties [13–15]. Thus recent research have incorporated more complicated tissue microstructure models into DWI data analysis to better provide estimates of specific tissue properties and explain the detailed diffusion signal decay, attempting to model the diffusion signal in tumors exist [16–18].

Among these multicompartmental models, the incoherent motion model (IVIM) by Le Bihan et al. [19] uses biexponential curve fitting to assume that tissue water resides in two non-exchanging compartments: vascular (pseudo-diffusing water inside blood vessels) and nonvascular (diffusing water in and around cells). Further, Bennett et al. [20] introduced the stretched-exponential model (SEM) to assess intravoxel diffusion heterogeneity by measuring the distributed diffusion coefficient and the diffusion heterogeneity index α. In addition, the restriction spectrum imaging (RSI) model, an advanced linear mixture model deconstructs the DWI signal into namely restricted, hindered, and free water pools, allowing different perspectives for analysis of water molecule behavior [21]. These models have shown promise in differentiating benign from malignant breast tumors and in evaluating Ki-67 expression level [22, 23].

Previous model likes IVIM separates out the additional signal from vascular water, its description of diffusion in the cellular component of the tissue remains simple monoexponential decay. It does not account for cellular compartmentalization and restriction, anisotropy, or other biophysical effects that are found in breast tumors [24, 25]. The multimodal apparent diffusion (MAD) is a multi-compartment model proposed by Damen [26], is an extension of multi-exponential analysis combined with the SEM. It characterizes water diffusion in tissues by separating the diffusion signal into four distinct components: flow (pseudo-diffusing water in blood vessels), unimpeded (fluid), hindered (delayed passage of molecules navigating cellular obstacles), and restricted diffusion (water molecules trapped within cell membrane). Variations in signal intensity across voxels are postulated to stem from changes in the relative dimensions of these intravoxel water compartments.

Recently, MAD model has been preliminarily investigated in brain diseases by Damen et al. [26]. The application of MAD in breast cancer is particularly challenging because of the complex breast tissue microstructure and cancer heterogeneity, which resulting that there is still no relevant studies and this is a relatively novel model. The aim of this work was to extend the MAD framework for modelling breast cancer, enabling comprehensive characterization of tumoral regions, with a particular emphasis on cellular and vascular characteristics. The content of this study was to explore the application of MAD parameters in the diagnosis of benign and malignant breast lesions and in the prediction of Ki-67 expression in breast cancer. These preliminary results hold promise for the non-invasive characterization of breast tumors to decrease the number of excessive biopsies by MAD model, which would be an important tool for diagnosis and monitoring of treatment effects.

## Materials and methods

### Patients

This retrospective study, approved by the ethics committee of local institution, included 112 female patients (from July to November 2022) suspected of having breast lesions based on clinical palpation, ultrasonography, or mammography. The requirement for the informed consent was waived due to the study's retrospective nature.

The inclusion criteria were as follows: absence of contraindication to MRI examinations; MRI scans conducted no more than two weeks prior to surgery intervention; lesions histopathological confirmed post-surgery; no previous biopsy and anti-tumor treatment; and image quality approved as satisfactory (good image resolution, good lesion conspicuity from the surrounding normal tissue, and high SNR; absent distortion and artifacts). Exclusion criteria encompassed: nonoptimal DWI images due to motion and susceptibility artifacts (*n* = 2); lack of histopathologic confirmation (*n* = 3); absence of detectable lesion on MRI image (*n* = 5); prior breast cancer treatments (*n* = 3); and lesions with excessive necrosis or hemorrhage (*n* = 6). Ultimately, the study analyzed 93 lesions (90 patients) from the initial cohort, and 3 were confirmed to have bilateral breast lesions of the breast. All of them were pathologically confirmed through surgical procedures. It was clear that MRI-MAD scan was done in all cases for clinical purposes. All evaluated lesions were subsequently surgically excised and underwent postoperative pathologic confirmation.

### MRI data acquisition

All MRI scans were performed on a 3.0 T scanner (uMR 790, United Imaging Healthcare, Shanghai, China) equipped with a dedicated bilateral breast coil with 10 channels. All participants were positioned prone, without breast compression. The DWI in axial view was executed prior to contrast agent injection, and utilized 16 b values (0, 10, 20, 30, 50, 70, 100, 150, 200, 400, 800, 1200, 1500, 2000, 2500, and 3000 s/mm^2^). The corresponding number of excitation is 1, 1, 1, 1, 1, 1, 1, 1, 1, 1, 2, 2, 3, 6, 7, and 7. The detailed parameters were as follows: TR/TE = 3800/66.4 ms, field of view = 190 mm × 350 mm, acquisition matrix = 104 × 192, slice thickness = 4.0 mm, 28 slices, total scan time = 7 min 10 s. Additional breast MRI sequences included: 1) T1-weighted axial fast spin echo (FSE); 2) T2-weighted axial fat suppression FSE; followed by 3) dynamic contrast enhanced (DCE) MRI sequences. All MRI sequence parameters are shown in Table 1.

**Table 1:** Imaging Protocol Parameters for T1WI, T2WI, DCE-MRI, and MAD^a^

	T1WI	T2WI	DCE-MRI	MAD
Sequence	FSE	FSE_SPAIR	GRE_QUICK	EPI_DWI
Orientation	Axial	Axial	3-dimension	Axial
TR (msec)	607	2440	4.86	3800
TE (msec)	7.8	86.5	2.23	66.4
FOV (mm × mm)	340 × 340	340 × 340	340 × 340	190 × 350
Matrix	648 × 648	496 × 552	498 × 624	104 × 192
Number of slices	28	28	-	28
Imaging time (min)	2:48	2:27	7:23	7:10
b-value (sec/mm^2^) (number of incentives)	-	-	-	0(1), 10(1), 20(1), 30(1), 50(1), 70(1), 100(1), 150(1), 200(1), 400(1), 800(2), 1200(2), 1500(3), 2000(6), 2500(7), 3000(7)

^a^ *DCE-MRI* dynamic contrast-enhanced MRI, *MAD* multimodal apparent diffusion, *FSE* fast spin echo, *EPI* echo-planar imaging, *FOV* field of view. "-", means not applicable

### Image analysis

All diffusion imaging data were processed using the ITK-Snap software (open-source; www.itk-snap.org) and MATLAB (MathWorks, Inc., Natick, MA) for post processing.

The study employed the MAD, a novel model with a predefined number of components linked to microstructure. This method, described as a quad-modal diffusion model [26], utilizes multivariate nonlinear regression to analyze diffusion-weighted signal decay in MRI scans across various b-values. The formula used is

$$\frac{S(b)}{S(0)}=f_{F}{\cdot}exp(-D_{R}\cdot{b})+f_{H}\cdot{exp}(-D_{H}\cdot{b^{\alpha_{H}}})+f_{UI}\cdot{exp}(-D_{UI}\cdot{b})+f_{F}\cdot{exp}(-D_{F}\cdot{b}).$$

This formula facilitates identifying distinct apparent diffusivity modes, with the parameters, ${f}_{F}$, ${f}_{UI}$, ${f}_{H}$ and ${f}_{R}$ denoting the fractions of flow (D >  > 3 μm^2^/ms), unimpeded (UI) diffusion (D = 3 μm^2^/ms), hindered (H) diffusion (D > 0.2 & < 3 μm^2^/ms), and restricted (R) diffusion (D < 0.2 μm^2^/ms), respectively. D_X_ represent the diffusion coefficients for these compartments. This approach enhances the characterization of tissue properties by minimizing the least squares difference between the model and data, and incorporating linear regression for increased efficiency and noise resilience. The diffusion coefficient for unimpeded diffusion D_UI_ is a universal constant that is approximately the same across different tissues because it represents the diffusion of water molecules that are not hindered by any cellular or structural barriers. This coefficient typically has a value around 3 × 10^−3^mm^2^/s^3^, representing the free diffusion of water in a homogeneous medium, such as pure water at body temperature.

The ADC derived from monoexponential DWI model was calculated for comparison using the following equation:

$$S/S_{0} = e^{b*ADC}$$

where S is the signal intensity acquired at b-values = 400, 800 s/mm^2^. S_0_ is the signal intensity in the voxel with b-value = 0 s/mm^2^.

Regions of interest (ROIs) were manually delineated on the largest slice of the DWI image at b = 800 s/mm^2^ by two independent radiologists (Z.Q.S, reader A, with 15 years of MR imaging experience; and C.H, reader B, with 2 years of MR imaging experience). Both of them analyzed all images independently and were blind to histopathologic outcomes. The two readers’ average values were used for the final analysis of diagnostic performance. The DCE images assisted the lesion localization and the boundary verification. Cystic components, necrotic areas, and hemorrhage areas were avoided. The ROI demarcation at b = 800 s/mm^2^ was chosen for its optimal contrast between lesions and surrounding tissue. The delineated ROI contours were then registered and transferred to the maps of f_F_, f_UI_, f_H_, f_R_, D_F_, D_H_, D_R_, α_H_, and calculated on a voxel-by-voxel basis. Registration ensured alignment of the ROIs across different imaging sequences, maintaining spatial accuracy. Mean value of MAD diffusion parameters was then computed from these ROIs, providing the primary measures for analysis.

### Histopathology analysis

All patients underwent mastectomy or lumpectomy, with the surgical specimens subsequently prepared for histological assessment. The final histopathological analysis of tumor specimens served as the reference standard. Ki-67 nuclear protein expression, indicative of cell proliferation, was quantified by the percentage of immunoreactive tumor cells. A high expression threshold was set at 20%, marked by positive immunostaining in tumor cell nuclei exceeding this value [27, 28].

### Statistical analysis

All statistical analyses were performed using SPSS (v. 19.0; Chicago, ILs). Interobserver reliability of MAD measurements was assessed by intra-class correlation coefficient, and Dice coefficiency for ROIs to ascertain reliability. Normality was assessed using the Shapiro-Wilks test. The Mann‒Whitney U test or Welch’s t test was used to compare the differences in MAD parameters between the benign and malignant breast lesion groups, as well as in the high Ki-67 and low Ki-67 expression groups. The binary logistic regression and receiver operating characteristic (ROC) curve were used to assess the diagnostic performances of the individual MAD parameters and their combinations. Sensitivity and specificity metrics were calculated based on the optimal cutoff points derived from the ROC curves using Youden's index. In addition, the area under the ROC curve (AUC), accuracy, sensitivity, and specificity were calculated, with AUC expressed as a mean and 95% confidence interval (CI). The AUCs were compared using the Delong test. Due to exploratory nature of the study, no correction for multiple comparisons was performed. *P*-values < 0.05 were taken to indicate statistical significance.

## Results

### Clinical characteristics

Of the 93 lesions in this study, 20 lesions were benign, and 73 lesions were malignant (Table 3). Among the 73 malignant lesions evaluated for Ki-67 expression, 42 lesions exhibited a Ki-67 level below 20%, categorizing them into the Ki-67 low expression group. In contrast, 31 lesions had a Ki-67 level exceeding 20% and were categorized in the Ki-67 high expression group. The clinical characteristics of the patients and lesions are presented in Tables 2 and 3.

**Table 2:** The patients and lesions’ characteristics^a^

	Benign(*n* = 20)	Malignant(*n* = 73)
**Patient characteristics**		
Age(years)^b^	40.5 (34, 46.5)	53 (42, 64)
Menstrual status^a^		
Premenopausal	16 (80)	33 (45.2)
Postmenopausal	4 (20)	40 (54.8)
**Lesion characteristics**		
Size(range, mm)^b^	14.5 (10, 20.25)	20 (13, 26)
Lesion type^a^		
Mass	20 (100)	66 (90.4)
Non-mass	0 (0)	7 (9.6)
BI-RADS^a^		
3	4 (20)	0 (0)
4a	5 (25)	3 (4.1)
4b	5 (25)	19 (26.0)
4c	6 (30)	51 (69.9)
Kinetic curve type^a^		
Persistent enhancement	0 (0)	2 (2.7)
Plateau	20 (100)	65 (89.0)
Washout	0 (0)	6 (8.2)

^a^Data are presented as n (%). ^b^Data are presented as medians (interquartile ranges)

**Table 3:** The pathological characteristics of benign and malignant lesions^a^

	Number
Benign lesions^b^	20
Fibroadenoma	6 (30)
Fibrosis hyperplasia	6 (30)
Intraductal papilloma	4 (20)
Benign lobular tumor	3 (15)
Granulomatous lymphadenitis	1 (5)
Malignant lesions^b^	73
IDC	65 (89.0)
ILC	2 (2.7)
DCIS	6 (8.2)
Molecular prognostic factors^b^	
Ki-67	
≥ 20%	31 (42.5)
< 20%	42 (57.5)
ER	
Positive	51 (69.9)
Negative	22 (30.1)
PR	
Positive	39 (53.4)
Negative	34 (46.6)
HER-2	
Positive	15 (20.5)
Negative	58 (79.5)
Grade	
1	12 (16.4)
2	42 (57.6)
3	19 (26.0)

^a^ *ER* estrogen receptor, *PR* progesterone receptor, *HER-2* human epidermal growth factor receptor 2, *IDC* invasive ductal carcinoma, *ILC* invasive lobular carcinoma, *DCIS* ductal carcinoma in situ

^b^Data are presented as n (%)

### Inter-reader reliability of MAD-derived parameters measurements

The overall Dice similarity coefficient across all lesions was 0.86 (95% CI, 0.79–0.96). The ICC with 95% CI for the representative values of MAD parameters are shown in Supplementary Table 1.

### Comparative analysis of MAD parameters in benign/malignancy

**Fig. 1:**
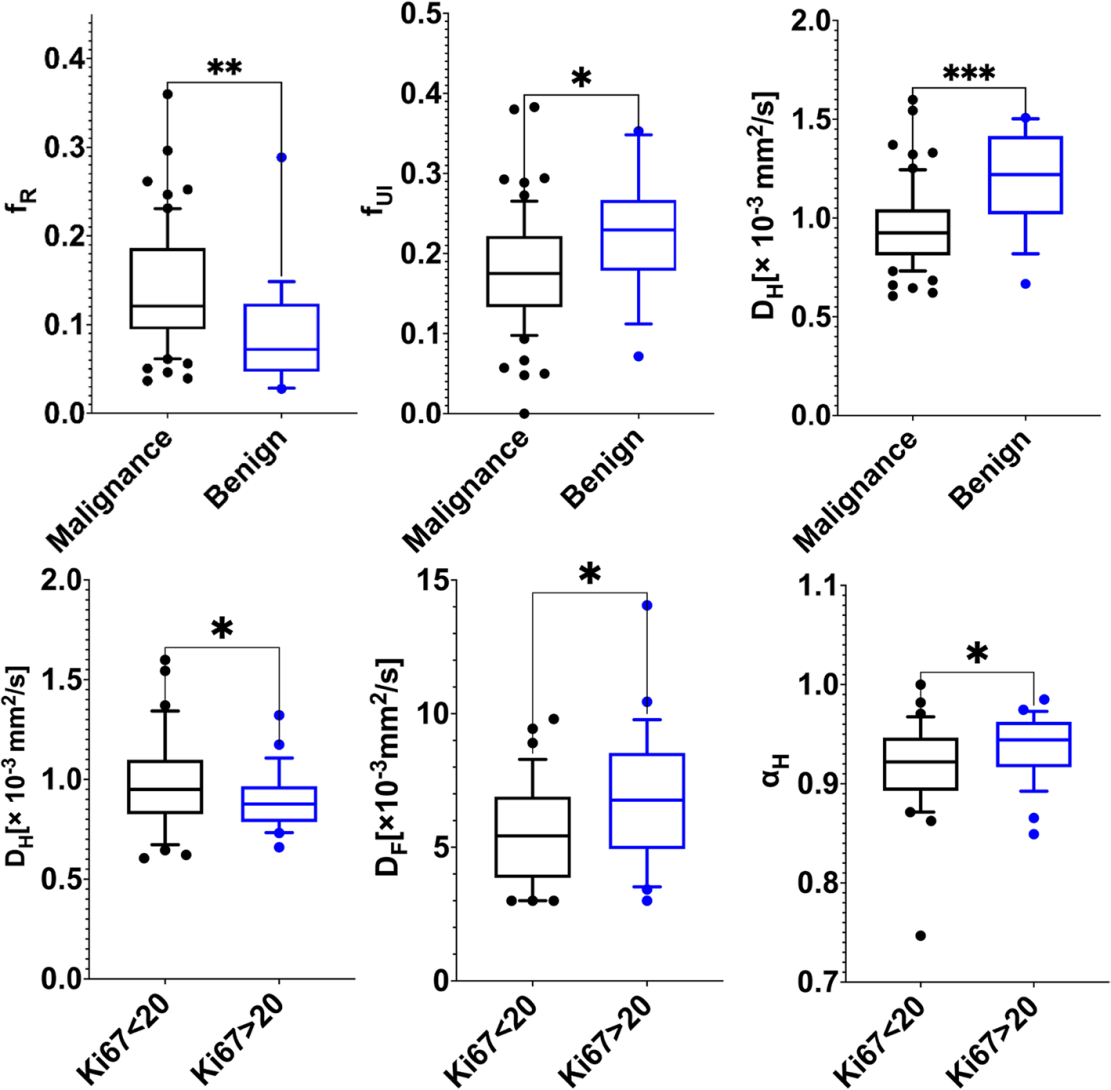
The top row of boxplots show f_R_, f_UI_, and D_H_ in benign and malignant lesions; The bottom row of boxplots show D_H_, D_F_, and α_H_ in high Ki-67 and low Ki-67 groups

**Fig. 2:**
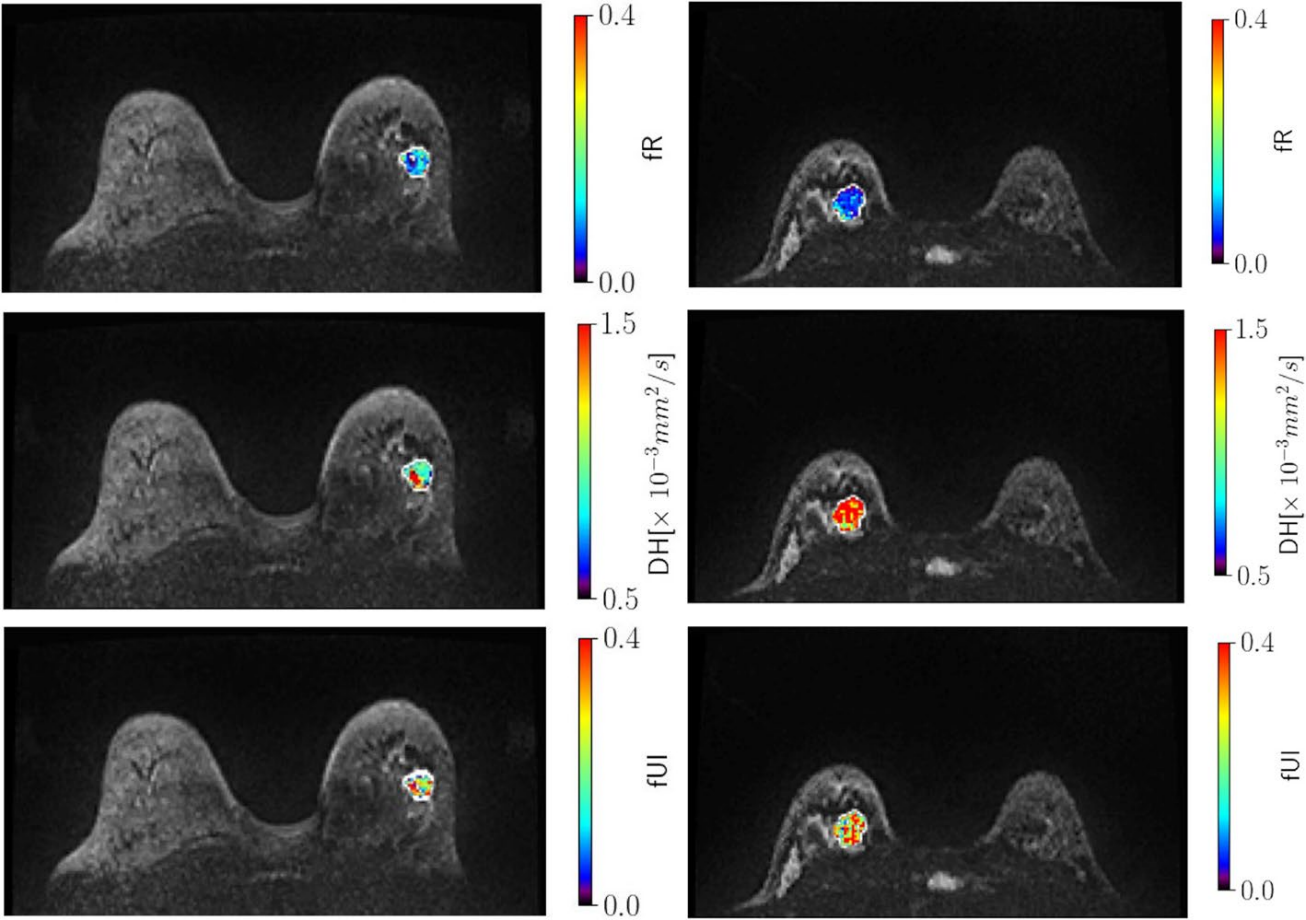
The maps of MAD-derived parameters f_R_, D_H_, and f_UI_ in the discrimination of benign / malignant lesions. The left column shows a 55-year-old woman with invasive ductal breast cancer. The right column shows show a 36-year-old woman with benign lobular tumor of the breast. The ROI was indicated by the white contours

The descriptive statistics of the MAD parameters and the *P* values are summarized in Table 4. The f_R_ in the malignant lesions was significantly higher than that in the benign lesions (0.140 ± 0.0668 *vs*. 0.091 ± 0.063, *P* = 0.001) (Figs. 1 and 2). The f_UI_ and D_H_ were found to be significantly lower in the malignant lesions compared with the corresponding values in the benign lesions (f_UI_: 0.178 ± 0.071 *vs*. 0.228 ± 0.077,* P* = 0.007, D_H_: 0.947 ± 0.205 μm/mm^2^ *vs*. 1.198 ± 0.246 μm/mm^2^,* P* < 0.001, respectively) (Figs. 1 and 2). Other MAD parameters did not show significant differences between the groups. The ADC in the malignant lesions was significantly lower than that in the benign lesions (1.078 ± 0.254 × 10^–3^ mm^2^/sec *vs*. 1.418 ± 0.296 × 10^–3^ mm^2^/sec, *P* < 0.001).

**Table 4:** Comparisons of MAD parameters and ADC among benign / malignant lesions and high/low Ki-67 expression levels^a^

parameter	Breast lesions		*P* value	Ki-67 status		*P* value
	Malignant (*n* = 73)	Benign (*n* = 20)		High (*n* = 30)	Low (*n* = 40)	
f_R_	0.121(0.095–0.187)	0.072(0.047–0.124)	0.001*	0.148 ± 0.078	0.134 ± 0.057	0.433
f_H_	0.638 ± 0.102	0.630 ± 0.069	0.664	0.635 ± 0.081	0.641 ± 0.118	0.834
f_UI_	0.178 ± 0.071	0.228 ± 0.077	0.017*	0.172(0.139–0.207)	0.182 ± 0.078	0.558
f_F_	0.042(0.023–0.057)	0.052 ± 0.035	0.260	0.042(0.024–0.057)	0.042(0.022–0.570)	0.877
D_R_	0.103 ± 0.051	0.093(0.050–0.118)	0.499	0.106 ± 0.042	0.101 ± 0.057	0.605
D_H_	0.925(0.811–1.045)	1.198 ± 0.246	< 0.001*	0.901 ± 0.148	0.984 ± 0.236	0.046*
D_F_	6.240(4.048–7.757)	6.665(3.796–8.041)	0.718	6.817 ± 2.483	5.610 ± 1.931	0.025*
α_H_	0.931(0.907–0.959)	0.919 ± 0.0516	0.712	0.937 ± 0.033	0.922(0.893–0.947)	0.034*
ADC	1.078 ± 0.254	1.418 ± 0.296	< 0.001*	1.131 ± 0.277	1.008 ± 0.205	0.035*

^a^ *ADC* apparent diffusion coefficient. *P-value less than 0.05

### Comparative analysis of MAD parameters in Ki-67 expression

The descriptive statistics of the MAD parameters and the *P* values are summarized in Table 4. Malignant breast lesions with Ki-67 high expression showed a significantly lower D_H_ compared to that with Ki-67 low expression (0.901 ± 0.148 μm/mm^2^ *vs*. 0.984 ± 0.236 μm/mm^2^, *P* = 0.046). The significant higher D_F_ and α_H_ were observed in the Ki-67 high expression group (*P* = 0.025, *P* = 0.034, respectively) (Fig. 1). No significant differences were observed in other MAD parameters between high and low Ki-67 expression groups. The ADC in the Ki-67 high expression group was significantly lower than that in the Ki-67 low expression group (1.008 ± 0.205 × 10^–3^ mm^2^/sec *vs*. 1.131 ± 0.277 × 10^–3^ mm^2^/sec, *P* = 0.035).

### ROC analysis among the individual and combination parameters

**Fig. 3:**
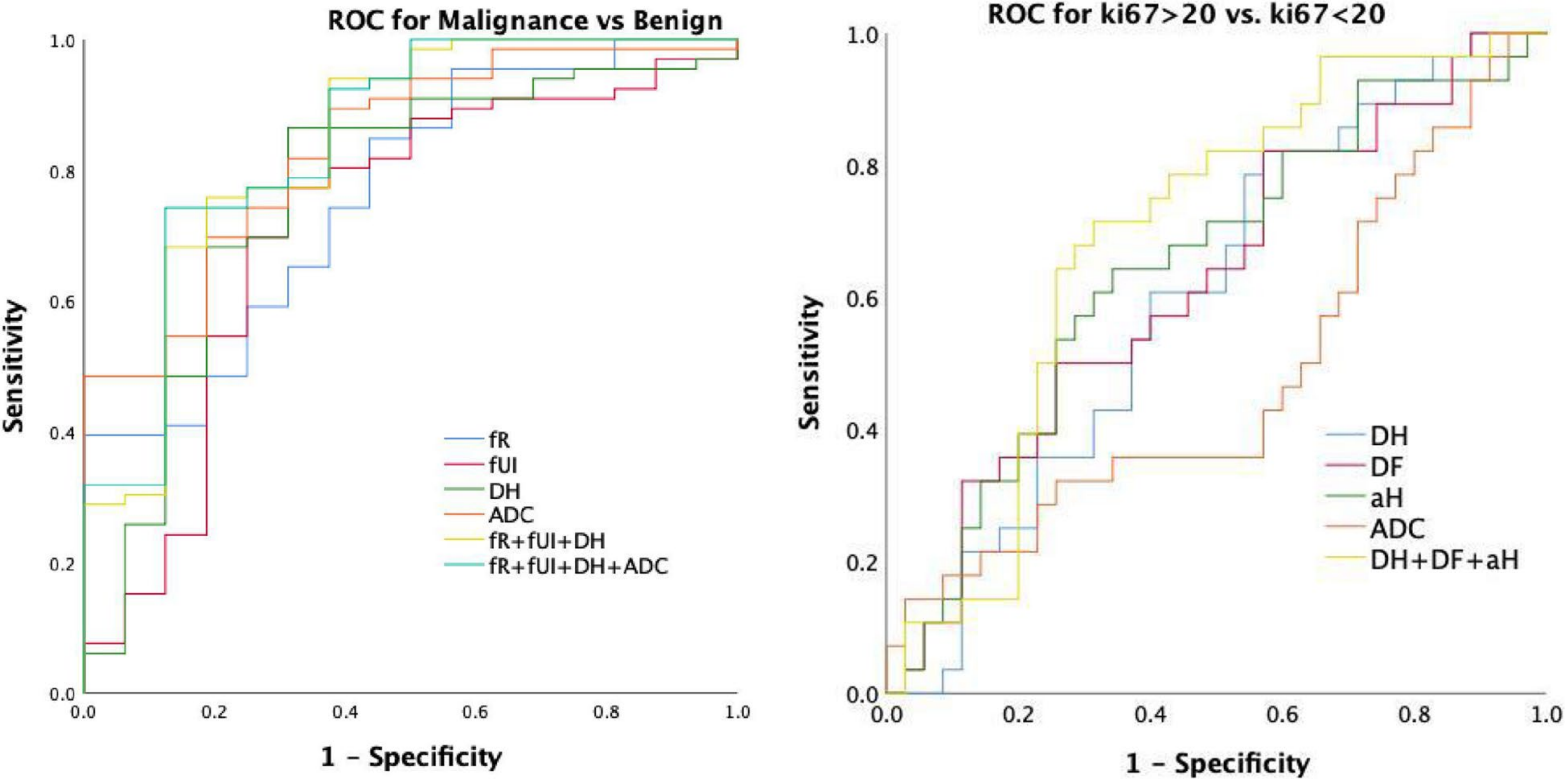
The graph shows ROCs to assess utility of MAD-parameters and ADC for discriminating malignant and benign lesions (left). The graph shows ROCs to assess utility of MAD-parameters and ADC for discriminating high and low Ki-67 expression levels (right)

The Table 5 and Fig. 3 presented the ROC analysis results of the f_R_, f_UI_, D_H_ and ADC in differentiating malignant from benign breast lesions. Of the single parameter, ADC achieved the highest AUC of 0.826. Regarding of the MAD parameters, the combination of f_R_, D_H_ and f_UI_ yielded the highest AUC of 0.851.

**Table 5:** Receiver operating characteristic analysis of ADC, and MAD-derived parameter in the discrimination of benign / malignant lesions^a^

Parameter	AUC (95% CI)	Cutoff	Sensitivity	Specificity	Accuracy(%)
f_R_	0.738(0.597–0.878)	0.078	0.859	0.611	78.0
f_UI_	0.697(0.545–0.849)	0.204	0.703	0.722	78.0
D_H_	0.775(0.641–0.909)	1.228 $\times$ 10^–3^	0.891	0.667	81.7
ADC	0.826(0.706–0.946)	1.241 $\times$ 10^–3^	0.786	0.778	78.4
f_R_ + f_UI_ + D_H_	**0.851(0.750–0.951)**	0.661	0.889	0.688	86.6

^a^ *CI* confidence interval, *ADC* apparent diffusion coefficient. The highest AUC value in the discrimination of benign/malignant lesions is shown in bold

The Table 6 and Fig. 3 presented the ROC analysis results of the D_H_, D_F_, α_H_ and ADC in the evaluation of Ki-67 expression levels. For the single parameter, the ADC produced the highest AUC of 0.648. For the multiple parameters, the combination parameters of MAD (D_H_, D_F_, and α_H_) demonstrated slightly higher AUC of 0.691 than ADC.

**Table 6:** Receiver operating characteristic analysis of ADC, and MAD-derived parameter in the discrimination of Ki-67 status^a^

Parameter	AUC (95% CI)	Cutoff	Sensitivity	Specificity	Accuracy(%)
D_H_	0.610(0.472–0.748)	1.041 $\times$ 10^–3^	0.821	0.444	59.4
D_F_	0.605(0.466–0.745)	6.932 $\times$ 10^–3^	0.500	0.722	56.3
α_H_	0.644(0.506–0.782)	0.934	0.643	0.667	59.4
ADC	0.648(0.514–0.783)	1.032	0.600	0.700	65.7
D_H_ + D_F_ + α_H_	**0.691(0.560–0.823)**	0.457	0.714	0.694	67.2

^a^ *CI* confidence interval, *ADC* apparent diffusion coefficient. The highest AUC value in the discrimination of Ki-67 status is shown in bold

## Discussion

In this study, we assess the MAD model’s application of diagnosing breast cancer and detecting tumor proliferation level. The current study showed that the parameters D_H_, f_R_, and f_UI_ were statistically different between malignant and benign breast lesions. MAD parameters can predict the proliferation level of breast cancer in addition to identifying benign and malignant breast lesions, and the parameters D_F_, D_H_, and α_H_ significantly differentiated high and low Ki-67 expression in breast cancer. These results suggested the MAD model can be used to obtain more detailed information about water diffusion and tissue microstructure in breast tumors.

In the present study, D_H_ was lower in the malignant lesions than in the benign ones. The hindered apparent diffusion component is normally assumed to the hindered diffusion of free water molecular colliding with cellular borders, indicating hindered water movement in the extracellular space. This decrease in extracellular space is attributed to the abnormal proliferation of cancer cells [29]. Such changes in cellular and micro-vessel density, coupled with the disorder of fibrous tissue, are likely to restrict water molecules diffusion in extracellular space [30]. These structural changes in cancerous lesions provide a plausible explanation for the decrease in D_H_. It also suggests that MAD analysis can isolate water molecular diffusion signal in the extracellular environment of malignant lesions. Sigmund et al. [24] reported that the diffusion coefficient of the slow diffusion component obtained with biexponential analysis was influenced by tissue cellularity and tended to be lower in malignant breast lesions compared to normal fibrous tissue. Ohno et al. [31] demonstrated that slow-restricted diffusion obtained with triexponential analysis was significantly higher in ductal carcinoma in situ (DCIS) than in invasive ductal carcinoma (IDC).

Additionally, malignant lesions exhibited not only a marked decrease in D_H_, but also a significant increase in f_R_, which reflects water molecules movement in cells. The f_R_, defined as the fraction of diffusion signal from water confined within cellular compartments, may represent restricted water due to the presence of tissue microstructures, such as cell membranes and myelin [32, 33]. This change in cell membranes and myelin was noted in a study of brain [34]. It was found that a more pronounced restriction of intracellular water diffusion in malignant lesions was associated with some factors, such as the intracellular macromolecular crowding, increased viscosity of the cellular membrane, and reduced permeability of the cell membrane and myelin sheath. White et al. [35] hypothesized that the extent of this restricted diffusion is influenced by both cellularity and nuclear volume fraction of individual cells in triexponential model analysis. In Damen et al.’s study of MAD model for gliomas diagnosis, the restricted diffusion was found in the rim of the glioblastoma, aligning with areas of high cellularity within these solid tumors [26]. Additionally, the study found that f_UI_ was lower in malignant breast lesions than that in the benign lesions. The parameter f_UI_ characterizes unimpeded diffusion, possibly relating to the Brownian motion of water molecular in the extracellular space. This is consistent with the complex, heterogeneous intercellular environment of breast cancer. In malignant lesions, the cell density of the lesion is higher, with a small cell gap and the microstructure is more heterogeneous due to the uncontrolled proliferation of the cancer cells. Free movement of water molecular in the interstitium is restricted by irregularly proliferating cancer cells.

Ki-67 is an indicator of cell proliferative nature, with breast cancer exhibiting higher Ki-67 expression often characterized by hypercellularity, nuclear enlargement and atypia [2, 36]. The Ki-67 status serves as a predictor for pathological complete response before neoadjuvant chemotherapy, being associated with high risk for metastasis or recurrence, poorer prognosis, and reduced survival, thus marking more aggressive forms of breast cancer [37, 38]. In this study, the D_H_ was significantly lower in the Ki-67 high expression group than that in the Ki-67 low expression group. This observation aligns with the characteristics of the Ki-67 high expression group, which typically displays highly proliferative tumors with denser cell arrangement and reduced extracellular space, leading to more significant hindrance of water molecules [39]. This distinction in D_H_ could be instrumental in identifying more aggressive types of breast cancer.

Furthermore, the D_F_ was observed to be slightly higher in the Ki-67 high expression group. D_F_, a diffusion coefficient of fast diffusion, represents the least restricted diffusion such as pools of fluid or flow through blood vessels. High expression of Ki-67 is closely associated with rapid proliferation of tumor cells and vascular permeability of tumors, resulting in higher microperfusion and lesser diffusivity [40]. We speculate that this can be explained by the fact that angiogenesis leads to differences in vessel density, permeability, and vessel volume. Tumor neovascularization is mostly immature vascular endothelium with large endothelial gaps and vascular permeability, which makes it easy for fluid to seep from the endothelium into the tissue interstitial space, resulting in an increase in total extravascular, extracellular fluid volume and higher D_F_ values than expected [41, 42]. Notably, the flow parameter is a local characteristic, indicative of diffusivity in adjacent tissue and overall blood flow within a voxel, and aligns closely with the global perfusion parameter. This alignment allows for meaningful comparisons in perfusion analysis. This is consistent with previous studies indicating increased perfusion in breast cancer with Ki-67 high level [22, 25]. In addition, α_H_ reflects the heterogeneity in voxels, with a range of 0–1. Smaller α value indicates greater the heterogeneity of water molecular diffusion [43]. Compared with Ki-67 high status, Ki-67 low status has smaller cell density and looser extracellular interstitium, where the diffusion process of water molecular is more complex and various. The smaller α_H_ emphasize that the water diffusion environment is more heterogeneous in the Ki-67 low expression cancers.

In the current study, for the diagnosis of breast cancer and the assessment of Ki-67 expression, the individual MAD derived parameter shows significant differences. Although the diagnostic efficacy was slightly improved compared MAD parameters to ADC, the elevation is not statistically different. Additionally, it also can be observed in this study that the diagnostic efficacy of the combination multiple parameters of MAD is higher than that of a single parameter. This demonstrates to some extent the advantage of multiparameter, not only reflecting the real dispersion characteristics of water molecules inside and outside the cell, but also the flow of water molecular in the tissue, as well as the heterogeneity of water molecular dispersion in the tissue, enabling us to understand the diverse diffusion patterns of water molecules in breast cancer. For implementation of the MAD model, a current challenge is the long time needed for both acquisition and image processing. The study chooses a b-value range from 0 to 3000 s/mm^2^ in breast cancer imaging because it provides optimal differentiation between benign and malignant tissues, crucial for accurate diagnosis and treatment planning. Lower b-values (close to 0) capture tissue structure and perfusion, while higher b-values (up to 3000) reflect true diffusion properties, highlighting the restricted diffusion characteristic of malignant tumors. Empirical studies have shown that using b-values up to 3000 s/mm^2^ significantly improves the specificity and sensitivity of DWI in breast cancer detection [39, 44]. This range also maintains a good SNR (At the highest b-value the signal intensity is still greater than 20), ensuring high-quality images necessary for reliable diagnosis. Moreover, the technological capabilities of modern MRI scanners support the acquisition of high b-value images efficiently, making this range a practical choice for routine clinical use. The next step will focus on optimizing the method for clinical standards with a shorter protocol and applying it to larger patient cohorts. There is still much room for continued exploration in applying MAD to clinical diagnosis, such as the data fitting and analysis, and the current study is only an exploratory preliminary application of this concept.

This study had several limitations. Firstly, the current study focused more solely on the MAD diffusion parameters in the diagnosis of breast cancer, and did not compare it with standard BI-RADS lesion characteristics to show the additional value of DWI and MAD. Further studies could evaluate the diagnostic efficacy and applicability of MAD in the combination with imaging diagnostic features. Secondly, quantitative parameters were measured by averaging all voxels within the ROI. Yet, this approach offers essential initial insights and lays the groundwork for more intricate future analyses, such as histogram and texture analyses [45]. Moreover, the exploratory nature of this study involved multiple comparisons without correction, which increases the risk of Type I errors. Although the initial analysis aimed to identify potential significant parameters, future research should include corrections for multiple comparisons, such as the Bonferroni or False Discovery Rate adjustments, to ensure the robustness of the findings. Finally, the potential issue is overfitting of the model, especially relevant given the lower SNR in breast tissue, which requiring further optimization of the model’s fitting and analysis. Future research will involve using larger and more diverse datasets, employing cross-validation techniques, and applying model regularization methods to enhance robustness and generalizability. This presents an opportunity for broader, multi-institutional research to further validate and enhance the model.

## Conclusion

This study presents the MAD acquisition and mathematical model for noninvasively estimating microstructural characteristics in breast cancer, enabling comprehensive characterization of tumoral regions, with a particular emphasis on cellular and vascular characteristics. In conclusion, the MAD parameters are helpful in diagnosing breast cancer, and can be used for the preoperative prediction of Ki-67 status in breast cancer.

## Supplementary Information

Supplementary Material 1

## Data Availability

The datasets generated or analyzed during the study are available from the corresponding author on reasonable request.

## Abbreviations

DWI: Diffusion weighted imaging
MRI: Magnetic resonance imaging
ADC: Apparent diffusion coefficient
MAD: Multimodal apparent diffusion
FSE: Fast spin echo
DCE: Dynamic contrast enhanced
ROI: Region of interest
ICC: Intra-class correlation coefficient
ROC: Receiver operating characteristic
AUC: Area under the curve
CI: Confidence interval
DCIS: Ductal carcinoma in situ
IDC: Invasive ductal carcinoma
SEM: Stretched-exponential model
RSI: Restriction spectrum imaging

## Data availability

No datasets were generated or analysed during the current study.
