# Supplementary material for: Multimodal apparent diffusion MRI model in noninvasive evaluation of breast cancer and Ki-67 expression
Source: Cancer Imaging. 2024 Oct 11;24:137. doi: 10.1186/s40644-024-00780-x (PMC11470582; doi:10.1186/s40644-024-00780-x)
Supplement: Supplementary file 1 — Supplementary Material 1 [file 40644_2024_780_MOESM1_ESM.docx]

**Supplementary Table 1:** ICC for the MAD parameters measured by two radiologists

| parameters | ICC（lower bound, upper bound） | *F* |
| --- | --- | --- |
| f_R_ | 0.993 (0.988, 0.996) | 149.714 |
| f_H_ | 0.980 (0.965, 0.988) | 49.371 |
| f_I_ | 0.983 (0.970, 0.990) | 57.525 |
| f_F_ | 0.982 (0.969, 0.989) | 54.742 |
| D_R_ | 0.991 (0.985, 0.995) | 113.102 |
| D_H_ | 0.974 (0.956, 0.985) | 39.064 |
| D_F_ | 0.978 (0.962, 0.987) | 45.802 |
| α_H_ | 0.978 (0.962, 0.987) | 44.786 |

Abbreviations: ICC=intraclass correlation coefficient.
